# Effect of Exercise-Induced Muscle Damage on Bowling-Specific Motor Skills in Male Adolescent Cricketers

**DOI:** 10.3390/sports9070103

**Published:** 2021-07-19

**Authors:** Kenji Doma, Anthony Leicht, Carl Woods, Drew Harrison, Teneale McGuckin, Jonathan Connor

**Affiliations:** 1 Sport and Exercise Science, College of Healthcare Sciences, James Cook University, Townsville, QLD 4811, Australia; anthony.leicht@jcu.edu.au (A.L.); carl.woods@vu.edu.au (C.W.); drew.harrison@my.jcu.edu.au (D.H.); teneale.mcguckin@jcu.edu.au (T.M.); jonathan.connor@jcu.edu.au (J.C.); 2 Orthopeadic Research Institute of Queensland, Townsville, QLD 4812, Australia; 3 Institute for Health and Sport, Victoria University, Melbourne, VIC 3011, Australia

**Keywords:** strength training, muscle damage, delayed-onset muscle soreness, fast bowlers, youth sport

## Abstract

The current study examined the acute effects of a bout of resistance training on cricket bowling-specific motor performance. Eight sub-elite, resistance-untrained, adolescent male fast bowlers (age 15 ± 1.7 years; height 1.8 ± 0.1 m; weight 67.9 ± 7.9 kg) completed a bout of upper and lower body resistance exercises. Indirect markers of muscle damage (creatine kinase [CK] and delayed onset of muscle soreness [DOMS]), anaerobic performance (15-m sprint and vertical jump), and cricket-specific motor performance (ball speed, run-up time, and accuracy) were measured prior to and 24 (T24) and 48 (T48) hours following the resistance training bout. The resistance training bout significantly increased CK (~350%; effect size [ES] = 1.89–2.24), DOMS (~240%; ES = 1.46–3.77) and 15-m sprint times (~4.0%; ES = 1.33–1.47), whilst significantly reducing vertical jump height (~7.0%; ES = 0.76–0.96) for up to 48 h. The ball speed (~3.0%; ES = 0.50–0.61) and bowling accuracy (~79%; ES = 0.39–0.70) were significantly reduced, whilst run-up time was significantly increased (~3.5%; ES = 0.36–0.50) for up to 24 h. These findings demonstrate that a bout of resistance training evokes exercise-induced muscle damage amongst sub-elite, adolescent male cricketers, which impairs anaerobic performance and bowling-specific motor performance measures. Cricket coaches should be cautious of incorporating bowling sessions within 24-h following a bout of resistance training for sub-elite adolescent fast bowlers, particularly for those commencing a resistance training program.

## 1. Introduction

Cricket is an internationally popular sport involving repetitive, high intensity bouts of exercise, with bowling requiring the greatest anaerobic demands [1]. For example, it was suggested that bowling had similar demands to that of repeated-sprint exercise, manifesting in short bursts of running (i.e., a 15–20 m ‘run up’) with minimal recovery between deliveries (i.e., ~30 s) [2]. As a result, cricketers (primarily fast bowlers) commonly undertake resistance training to enhance physical attributes essential to cricket, such as muscular strength, power, and efficiency [1]. However, the eccentric component of resistance training can cause exercise-induced muscle damage (EIMD) for several days post-exercise [3], particularly for individuals unaccustomed to, or detrained from, this mode of training [4,5,6].

Common symptoms resulting from EIMD include muscle soreness, muscle stiffness and swelling, and a reduction of muscular contractility for several days post-exercise [7,8]. Following resistance training, EIMD has been reported to impair generic physical performance measures, including maximal isometric contractions [9,10], vertical jump [5], running economy [4,11], and cycling performance [12] for 24–48 h post-exercise. More recently, studies have reported attenuation in sport-specific performance measures, including skating performance in ice hockey players [13], repeated sprint ability in basketball players [14] and agility performance in volleyball players [15] during periods of EIMD. These findings suggest that EIMD may impair match-specific performance tasks and training quality [16], the reported EIMD was a result of match-simulated training sessions. However, match-simulated training may not replicate resistance exercises with external loads that localise physiological stress to particular muscle groups [17]. The acute physiological stress induced by resistance training, and its impact on sport-specific performance, may differ following resistance exercises (i.e., heavy external loads) compared to that of repeated anaerobic activities performed during a match (i.e., against bodyweight only). Further, EIMD may cause additional complications to sport-specific actions that have complex task-dynamics [18] (e.g., cricket bowling), relative to other, simpler actions, like sprinting and change of direction [13,15,19]. Complex coordinative patterns, such as cricket bowling, involve greater control of degrees of freedom, requiring greater demands of fine motor control and proprioceptive feedback [20]. However, the symptoms of EIMD are known to disrupt the sensory information necessary for movement perception and proprioception [21]. In fact, a recent study reported that skill learning during periods of EIMD were impaired using a dart throwing task [22], and the authors speculated that the disruption of the sensory feedback associated with EIMD symptoms may have hindered motor skill performance and learning. Thus, it is reasonable to assume that EIMD symptomatology may impair complex movement patterns to a greater degree than those that are considered more fundamental.

Recently, Doma et al. [23] reported a reduction in ball speed during a spiking protocol in collegiate volleyball players for 24-h following a bout of whole-body, resistance training (e.g., squats, deadlift, chest press) performed at 80% of one repetition maximum. Whilst these findings suggest that EIMD impairs sport-specific tasks, the findings may not be applicable to cricket, as the biomechanical demands of spiking are distinct to those of bowling. Further, the impact of EIMD may be exacerbated in those unaccustomed to resistance training, which may apply particularly to younger athletes who commence resistance training programs during adolescence [24]. The purpose of this study, then, was to examine the acute impact of EIMD, caused by resistance exercises, on bowling speed and accuracy in adolescent male fast bowlers. Identifying the impact of this common training modality on bowling-specific performance measures (e.g., bowling speed and accuracy) will improve understanding of the acute responses of this training and support the development of cricket athletes.

## 2. Materials and Methods

### 2.1. Participants

Eight sub-elite, adolescent male cricket fast bowlers (age 15 ± 1.7 years; height 1.8 ± 0.1 m; weight 67.9 ± 7.9 kg; 4.2 ± 2.5 years bowling) took part in the study via convenience sampling. The average peak bowling speed of the participants was 110.4 ± 12.5 km·h^−1^ and ranged from 102.5–118.2 km·h^−1^, which met the criterion required to be classified as a regional-level junior fast bowler according to Queensland Cricket. The participants were engaged in 30–60 min of bowling-specific training at least twice a week for the past 12-months but had not undertaken resistance exercises for the past 6-months. Each participant and their parent/guardian provided written informed consent prior to undertaking the study and did not report any acute or chronic illness, disease and injury or medication that would affect the testing protocols. All procedures were approved by the Institutional Human Research Ethics Committee (James Cook University, H6267) and were conducted in line with the Declaration of Helsinki.

### 2.2. Research Design

This study was conducted across three weeks. The first week consisted of a repetition maximum (RM) assessment session to determine 1 RM of incline leg press (AMF-656, APT Sports, South Australia, Australia), single-leg horizontal leg press (NS4000, Nautilus, Vancouver, WA, USA) and chest press (G9S, Body-Solid, Australia), and 6 RM for seated row (NS4000, Nautilus, Vancouver, WA, USA) and triceps extension (NS4000, Nautilus, Vancouver, WA, USA). The relative loading of RM testing was modified for each exercise to ensure safety during the test protocol. During the second week, there were three familiarisation sessions, which were separated by at least a day, and the participants were exposed to the bowling-specific test, anaerobic performance assessments and collection of indirect muscle damage markers in each familiarisation session. In the third week, baseline measures (Tbase) were collected in a single day, and assessments were conducted in the order of indirect muscle damage markers, anaerobic performance and bowling-specific test. The next day, a whole-body resistance training session was conducted to incur EIMD. The bowling-specific test, anaerobic performance and indirect muscle damage markers were reassessed at 24- (T24) and 48- (T48) hours following the resistance training session. These time points were selected, as the indirect markers of muscle damage typically peaks at 24–48 h following traditional resistance exercises involving various types of exercises in the one session [4,5,11].

### 2.3. Bowling-Specific Performance Test

**Figure 1:**
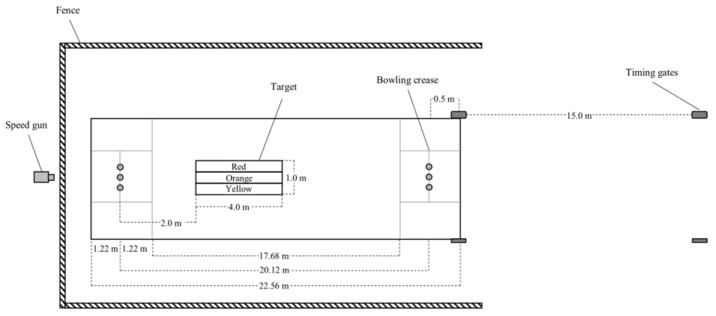
Schematic of the set-up for the bowling-specific performance test.

This test was conducted in a standard cricket practice net (Figure 1). Each participant undertook their bowling-specific training in this net during the season and were familiar with the facility. The participants completed three overs, consisting of six bowls/balls per over, with 30-s of rest in-between each bowl and three minutes of rest in-between each over. Given that three bowling-specific performance testing sessions were conducted within the same week, and that these protocols were conducted during periods of EIMD, the participants completed no more than three overs per session to minimise the risks of injuries. Further, the rest periods were implemented to replicate typical work:rest ratios for bowlers during a match [1]. The ball speed of each bowl was measured using a 100 Hz speed camera (Ultralyte UX 100LR, Colorado, CO, USA) situated behind the net. The average and peak ball velocity were calculated as the average and best values of the six bowls from the first (Ball*_avgV_*-Ov1 and Ball*_peakV_*-Ov1, respectively), second (Ball*_avgV_*-Ov2 and Ball*_peakV_*-Ov2, respectively), and third (Ball*_avgV_*-Ov3 and Ball*_peakV_*-Ov3, respectively) overs. The accuracy of bowling distance was determined visually using a target on the cricket pitch. Participants were instructed to bowl and hit a 1 × 4 m target placed longitudinally along the wicket, 2 m in front of the stumps (Figure 1). This position was selected to replicate a ‘good length’, which is a common target zone for bowlers to aim during training and a match [25]. The target was also demarcated horizontally from left to right into yellow-, orange-, and red-coloured zones, respectively, for accuracy of bowling direction (Figure 1). Participants were instructed to bowl at the coloured zones from left to right for overs one, two and three, respectively. Scoring for bowling accuracy consisted of three points when the ball landed within the correct coloured zone, two points when the ball landed within the adjacent coloured zone, one point when the ball landed within the coloured zone furthest away from the targeted coloured zone, and zero points when the ball landed outside the entire target area. Bowling accuracy was visually scored by two co-investigators, using a paper schematic of the target, who immediately and independently scored each bowl followed by discussion to confirm the final score for analysis (i.e., maximise inter-rater accuracy). Bowling accuracy was calculated as a percentage of the best score possible (i.e., 3 overs x 6 bowls x best score of 3 per bowl = 54, Accuracy-Avg) based upon the accuracy scores for the first (Accuracy-Ov1), second (Accuracy-Ov2) and third (Accuracy-Ov3) overs. Participants’ running speed per bowl was measured using an electronic timing gate system (Speedlight, Swiftperformance, Queensland, Australia) with the start line and finish line placed 15.5 m and 0.5 m away from the bowling crease, respectively (Figure 1). Similar to the bowling speed measures, the average and peak run-up completion times were calculated as the average and best of the six bowls for the first (Run*_avgT_*-Ov1 and Run*_peakT_*-Ov1, respectively), second (Run*_avgT_*-Ov2 and Run*_peakT_*-Ov2, respectively) and third (Run*_avgT_*-Ov3 and Run*_peakT_*-Ov3, respectively) overs. The run-up speed was recorded, given that a faster run-up speed results in a faster ball speed [26]. The Borg’s 6–20 rating of perceived exertion (RPE) was measured following each bowling delivery, which was then averaged for first (RPE-Ov1), second (RPE-Ov2) and third (RPE-Ov3) over and averaged across the three overs (RPE-Avg).

### 2.4. Anaerobic Performance and Sprint Tests

Anaerobic power was determined from sequential assessments of a countermovement jump (CMJ) and drop jump (DJ), whilst speed was assessed with a 15-metre sprint. For the CMJ test, participants completed three maximal jumps using a vertical jump apparatus (YardStick, Swift Performance, Queensland, Australia). During the eccentric component of the CMJ test, the participants were instructed to slightly flex at the knees and hips, hyper-extend the shoulders and maintain contact between the heels and the ground prior to take-off. During the concentric component, the participants were instructed to fully extend at the knees and ankles and swing their arms forward, using their dominant arm to tap the vanes of the vertical jump apparatus at peak jump height. For the DJ test, participants dropped from a 30 cm box and completed a CMJ (YardStick, Swift Performance, Queensland, Australia). Similar instructions were provided for the DJ, in addition to minimising the contact time between the feet and ground. Three trials were provided for the CMJ and DJ, respectively, with approximately 30-s of passive recovery given between each trial, and the best jump height used for later analyses. Finally, participants completed three, 15-metre sprints with two minutes of rest between each trial using the electronic timing gate system (Speedlight, Swiftperformance, Queensland, Australia). The best 15-m sprint time was recorded and used for later analyses.

### 2.5. Indirect Muscle Damage Markers

The indirect markers of muscle damage included creatine kinase (CK), delayed onset muscle soreness for the lower body (DOMS-LB), abdominals (DOMS-ABS) and upper body (DOMS-UB) and lower body range-of-motion using a sit-and-reach (S&R) test and hip horizontal abduction (ABD-ROM) test. The level of CK was assessed from a 30 μL fingertip, capillary blood sample collected from the non-bowling hand. The blood sample was immediately pipetted onto a test strip and assessed via a colorimetric assay (Reflotron, Boehringer Mannheim, Rotkreuz, Switzerland). The intra-assay coefficient of variation for CK within our laboratory was 7.2%, with the Reflotron system validated with good precision across several clinical laboratories [27]. Each DOMS measure was determined using the same 10-point visual analogue scale, with one denoted as “no soreness” and ten as “very, very sore” (Doma et al., 2015). For the DOMS-LB, participants performed one repetition of a body-weight squat, whilst one repetition of a sit-up and push-up exercise were performed for DOMS-ABS and DOMS-UB, respectively.

### 2.6. Repetition Maximum Assessment

Prior to the 1 RM test, participants completed a standardised warm-up of jogging on a treadmill for five minutes at a comfortable speed (i.e., 8–10 km∙h^−1^) followed by dynamic stretches of the upper and lower extremity (i.e., leg swings in the frontal and sagittal plane and arm swings in sagittal and transverse planes). Following the warm-up, sequential 1 RM tests were conducted for incline leg press, chess press and single-leg horizontal leg press, whilst 6 RM tests were conducted for seated rows and triceps extensions. The 1 RM and 6 RM tests were conducted in accordance with previously described methods [28]. Specifically, participants completed warm-ups sets of ten repetitions of each exercise at sub-maximal loads for incline leg press, chest press, single-leg leg press, seated rows and triceps extensions. Following the warm-ups sets, participants undertook 8–10 repetitions at near maximal workloads based on perceived effort using a 10-point visual analogue scale during the warm-up set. After 5-min of rest, loads were increased by 20–30% and 10–15% to attempt the 1 RM and 6 RM test, respectively, with final load achieved within 3–5 attempts, and each attempt separated by 5-min.

### 2.7. Resistance Training Session

The resistance training session was developed by a certified strength and conditioning coach with the intent of improving bowling-specific performance and consisted of all exercises performed in the same order as the RM testing session. Each exercise was performed at 70% of predetermined 1 RM (the predicted 1 RM for seated rows and triceps extensions were calculated from the 6 RM test) for three sets of 10 repetitions with approximately 1.5 min of rest between each set and exercise. For each set, participants rated their level of difficulty using a 10-point visual analogue scale ranging from one being “very easy” to ten being “very difficult”. If participants rated their level of difficulty below eight by the second set, then the load was increased by 5%. Conversely, if participants were unable to complete ten full repetitions, then the load was decreased by 5%. Finally, at the end of the resistance training session, participants completed an additional walking lunge exercise involving one dumbbell in each hand (i.e., approximately 30% of participant’s body mass with two dumbbells combined) to incorporate exercises that replicated movement patterns during running. For this exercise, participants completed three sets of lunges over 20-m per set and 1.5-min of rest between each set.

### 2.8. Statistical Analyses

The measure of central tendency and dispersion were reported as median and interquartile ranges. The outcome measures were compared between time points (i.e., Tbase vs. T24 vs. T48) using Friedman’s tests. Once a main time effect was identified, paired Wilcoxon tests were used to determine the location of differences. Given the small sample size in our study, and potential to violate the assumptions of the central limit theorem [29]. All data were analysed using the Statistical Package of Social Sciences (SPSS, IBM, version 25), with the alpha level set to 0.05. Effect sizes (Cohen’s d) were calculated to ascertain the magnitude of differences between the time points, with values of 0.2, 0.5 and 0.8 considered as small, moderate and large, respectively [30].

## 3. Results

### 3.1. Bowling Speed and Accuracy

The average and peak ball speeds were significantly reduced during the first and third overs (*p* < 0.05) and the overall average across the three overs (*p* < 0.05) at T24 compared to Tbase (Table 1). No significant differences were found for peak and average ball speeds during the second over (*p* > 0.05) at T24, and there were no differences for any of the ball speed parameters at T48 (*p* > 0.05). Bowling accuracy was significantly impaired at T24 compared to Tbase during the second over (*p* < 0.05). Compared to Tbase, there were no significant differences in bowling accuracy during first and third overs at T24 (*p* > 0.05), and no significant accuracy differences during any of the overs at T48 (*p* > 0.05). No differences were identified between T24 and T48 with respect to any of the bowling speed and accuracy measures (*p* > 0.05).

### 3.2. Run-Up Speed and RPE

The average run-up times were significantly greater at T24 and T48 than Tbase during the second over (*p* < 0.05), although the minimum run-up times were significantly greater at T24 than Tbase during the first and third overs (*p* < 0.05; Table 1). In addition, the overall average run-up times across the three overs were significantly greater at T24 and T48 (*p* < 0.05), whilst the average minimum run-up times were greater at T24 only (*p* < 0.05). Further, RPE averaged across the three overs were significantly greater at T24 (*p* = 0.05), although no differences were found at T48 (*p* > 0.05; Table 1). No differences were identified between T24 and T48 for any of the run-up speed and RPE measures (*p* > 0.05).

### 3.3. Anaerobic Power and Indirect Muscle Damage Markers

**Figure 2:**
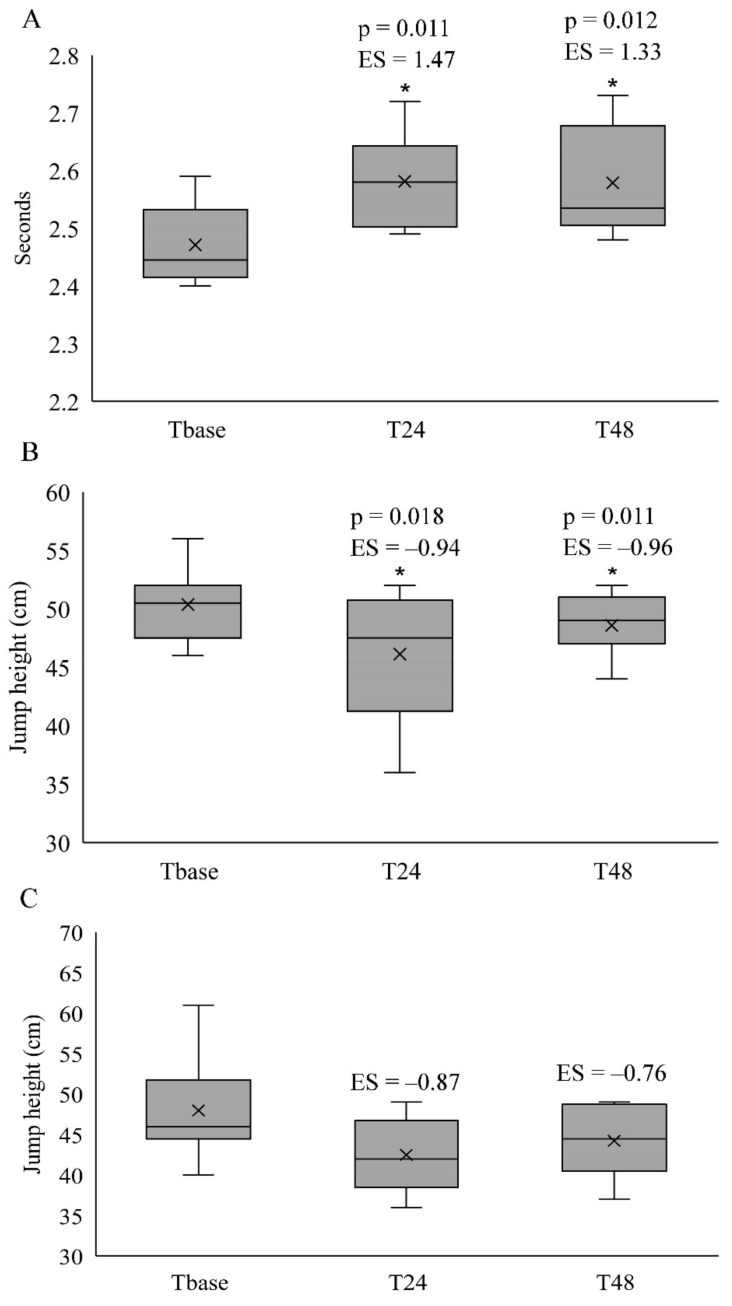
The median and interquartile ranges (25–75%) of the 15-m sprint times (**A**), countermovement jump height (**B**) and drop jump height (**C**) with *p*-values and effect size (ES) at T24 and T48 when compared to Tbase. * Significantly different from Tbase; cross symbol in the boxplots indicate mean values. Note: *p*-values have been provided only when the Friedman test exhibited a main effect time based on the paired Wilcoxon test, and ES have only been presented when a main effect of time was identified, or if the differences were moderate to large.

**Figure 3:**
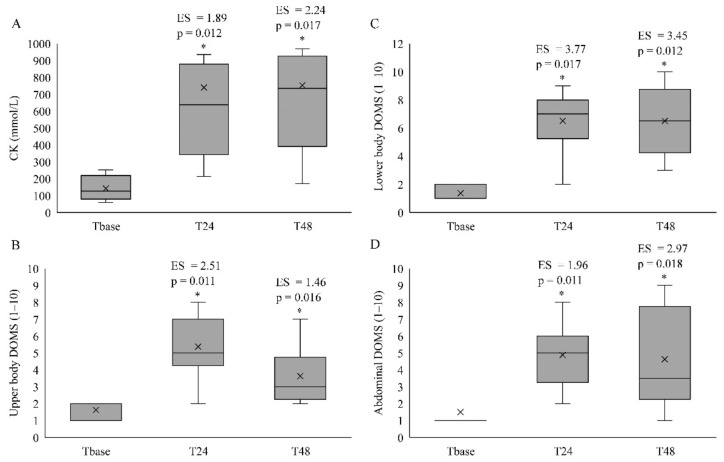
The median and interquartile ranges (25–75%) of creatine kinase (CK) (**A**), delayed onset muscle soreness (DOMS) of the upper body (**B**), lower body (**C**) and abdominals (**D**) with *p*-values and effect size (ES) at T24 and T48 when compared to Tbase. * Significantly different from Tbase; cross symbol in the boxplots indicate mean values. Note: *p*-values have been provided only when the Friedman test exhibited a main effect time based on the paired Wilcoxon test, and ES have only been presented when a main effect of time was identified, or if the differences were moderate to large.

The 15-m sprint times significantly increased (*p* < 0.05; Figure 2A), whilst CMJ significantly decreased (*p* < 0.05; Figure 2B) at T24 and T48 when compared to Tbase. However, no significant differences were identified for DJ measures between the time points (*p* > 0.05; Figure 2C). For the indirect muscle damage markers, CK (Figure 3A), DOMS-LB (Figure 3B), DOMS-UB (Figure 3C) and DOMS-ABS (Figure 3D) were significantly increased at T24 and T48 when compared to Tbase. The DOMS-UB was also significantly lower at T48 when compared to T24 (*p* = 0.016), although no differences were identified between T24 and T48 for any of the anaerobic power and indirect muscle damage markers (*p* > 0.05).

## 4. Discussion

This study showed significant reductions in overall bowling speed and longer run-up completion times for up to 24-h following the resistance training session. The bowling accuracy was also impaired for up to 24-h-post exercise, although this was only found during the second over, with no differences identified for the overall accuracy measure. Anaerobic power measures (i.e., 15-m sprint, CMJ) were impaired for up to 48-h following resistance training, whilst a number of indirect muscle damage markers (i.e., CK and DOMS) were elevated for up to 48 h following resistance training. These findings demonstrate that a bout of resistance training impaired bowling, run-up speed, and accuracy amongst adolescent fast bowlers. The attenuation in bowling-specific parameters may have been mediated by EIMD, and/or neuromuscular fatigue, caused by a traditional bout of resistance training, possibly due to compromises in muscle contractility, and ultimately leg power.

Directly comparing the current findings to other studies was difficult given that the acute effect of a resistance training bout on bowling-specific performance has not been examined previously, to our knowledge. Nonetheless, several studies have reported that a bout of traditional resistance exercises impaired a range of physical performance measures, including sub-maximal [4,5] and maximal running performance [6,10], cycling performance [12] and spiking ball speed in volleyball [23]. These studies also reported reductions in lower body, muscle force generation with increased DOMS, and suggested that compromised muscular contractility may in part have contributed to the attenuation in physical performance measures. The mechanisms for DOMS during periods of EIMD have been speculated to consist of activation of Group III and IV afferents caused by disruption of intermediate filaments, leakage of intra-muscular proteins, and compromised ability to maintain intramuscular calcium concentration, thereby impairing muscular function [31,32]. Given that the current study identified reductions in CMJ with elevated lower body DOMS, it was reasonable to assume that impaired bowling-specific performance may have been a result of the reduced lower body, anaerobic power caused by EIMD, and/or neuromuscular fatigue. In fact, lower body power as measured by jump height has been reported as a strong determinant of bowling speed amongst fast bowlers [33]. Subsequently, vertical jump performance may be a useful monitoring tool to assess athlete’s recovery and readiness for bowling following a bout of strenuous exercises during periods of EIMD.

Declines in run-up speed, and 15-m sprint ability were identified here, which likely contributed to the poorer bowling speed following a bout of resistance training. Other studies have reported similar attenuations in sprint performance during periods of EIMD [34,35]. Indeed, the run-up speed was considered essential to optimise ball speed and accuracy due to the combined transfer of momentum and summation of segmental velocities through the kinetic chain of the bowling action [26,36]. Interestingly, impaired bowling speed and accuracy was reported previously with a reduction in run-up speeds during hot and humid conditions [37]. Whilst these studies did not specifically examine the acute effects of a resistance training bout on bowling specific parameters, the authors suggested that fatigue accumulated from heat stress may have compromised bowling actions during the run-up, and ultimately impaired bowling-specific performance [38]. It was also suggested that athletes experiencing DOMS altered their running gait pattern in an attempt to limit the sensation of muscular discomfort [6,10]. Thus, the current bowlers may have adjusted their kinematics during the run-up to circumvent the symptoms of DOMS and impaired muscular contractility, which may have produced reductions in run-up speed. Further research examining the biomechanics of bowling performance during periods of EIMD (i.e., up to 48-h) will confirm altered gait as a contributor to impaired bowling performance during EIMD.

While potential changes in lower limb gait may have impacted bowling performance in the current study, high levels of DOMS within the core and upper body (i.e., DOMS-ABS and DOMS-UB) were identified. This indicates that body site-specific DOMS may have also contributed to the reduction in bowling speed and accuracy. Previously, muscle soreness was associated with impaired muscular contractility [6,9,10], with core strength a strong determinant of upper and lower body power output by optimising the generation and transfer of forces to the extremities [39]. In addition to core strength, studies have reported that upper body power, determined from bench press throw [33] and pull-up strength [40], was significantly related to bowling speed amongst fast bowlers, confirming upper body strength/power as an important fitness component for optimal bowling performance. Interestingly, DOMS in the upper body was the only muscle soreness measure that demonstrated greater recovery, with lower values at T48 when compared to T24. It is possible that the volume of upper body exercises performed during the resistance training bout resulted in this trend, given that fewer exercises were performed for the upper body. Nonetheless, DOMS in the upper body was still elevated for up to 48 h post-exercise. When coupled, our findings support the notion that DOMS within the core and upper body may contribute to a reduction in bowling speed and accuracy, possibly due to impaired contractility of the primary muscle groups for bowling.

## 5. Limitation

While the current study has demonstrated the significant impact of EIMD on bowling performance in adolescent fast bowlers, a number of limitations should be noted. Firstly, the bowlers recruited were adolescent males. Thus, findings may not be extrapolated to adult or female bowlers. However, given that resistance training is commonly introduced amongst adolescent athletes, examining its effect on bowling-specific performance measures was important for assisting with exercise prescription. Secondly, the bowling-specific performance measures constituted of only three overs, which is substantially less than a typical match. Three overs were selected for each testing session as recommended by the team physiotherapist to minimise the risks of injuries for adolescent bowlers. Nonetheless, given that bowling-specific performance measures were impaired during periods of EIMD within three overs, greater attenuation in these measures could be expected with a greater number of overs where fatigue likely accumulates across a match [2]. Further research is warranted to determine whether a bout of resistance training impairs bowling-specific performance across multiple overs replicating a cricket match. Third, we were unable to isolate whether the impairment of bowling-specific performance measures were a result of EIMD alone, and/or due to neuromuscular fatigue. Further research is necessary to determine whether neuromuscular fatigue possibly induced by a bout of resistance training impairs bowling-specific performance via peripheral and central pathways. Finally, given the repeated bout effect, it is unclear whether the observed effects in the current study would be replicated in those recently exposed to resistance training, whereby the level of EIMD and/or neuromuscular fatigue would be less evident.

## 6. Conclusions

This study demonstrated that a bout of traditional resistance training impaired bowling-specific performance for up to 48-h amongst adolescent fast bowlers. Reductions in lower limb neuromuscular function, impaired sprint-ability, and DOMS-induced changes in lower/upper body power were identified as possible contributors to the poorer performance following EIMD. Athletes and coaches should be cautious of incorporating bowling-specific activities (i.e., technical conditioning and match) during periods of EIMD that may limit performance and training adaptations. In addition, a recovery time of greater than 48-h is required for adolescent fast bowlers to minimise negative impacts on bowling-specific performance, particularly for athletes who are undertaking new resistance training regimes.

## Figures and Tables

**Table 1:** The median and interquartile ranges (25–75%) of the cricket-specific performance measures at baseline (Tbase), 24 h post-exercise (T24) and 48 h post-exercise (T48) during each over and the average of the three overs with *p*-values and effect size (ES) at T24 and T48 when compared to Tbase.

	Tbase	T24	T48
Averaged over six bowls in each over			
Ball*_avgV_*-Ov1 (km h^−1^)	101.6 (96.3–110.4)	98.9 (92.9–104.1); *p* = 0.012 *; ES = −0.61	102.0 (96.1–105.3); *p* = 0.208; ES = −0.32
Ball*_avgV_*-Ov2 (km h^−1^)	100.7 (95.0–107.8)	100.5 (95.2–103.5)	101.1 (96.2–104.2)
Ball*_avgV_*-Ov3 (km h^−1^)	100.9 (94.5–107.9)	97.0 (94.7–100.9); *p* = 0.025 *; ES = −0.50	101.1 (95.9–102.9); *p* = 0.484; ES = −0.14
Ball*_peakV_*-Ov1 (km h^−1^)	104.7 (99.1–114.1)	101.3 (96.7–105.8); *p* = 0.018 *; ES = −0.66	104.8 (98.2–109.4); *p* = 0.123; ES = −0.35
Ball*_peakV_*-Ov2 (km h^−1^)	102.5 (98.1–111.1)	102.4 (98.1–107.8)	104.8 (97.3–108.3)
Ball*_peakV_*-Ov3 (km h^−1^)	103.9 (97.6–112.4)	100.0 (98.2–103.6); *p* = 0.036 *; ES = −0.61	104.5 (100.6–107.4); *p* = 0.483; ES = −0.12
Run*_avgT_*-Ov1 (s)	2.76 (2.49–2.81)	2.84 (2.67–2.99)	2.81 (2.48–3.02)
Run*_avgT_*-Ov2 (s)	2.69 (2.51–2.49)	2.81 (2.60–3.00); *p* = 0.042 *; ES = 0.50	2.84 (2.49–3.03); *p* = 0.025 *; ES = 0.56
Run*_avgT_*-Ov3 (s)	2.67 (2.52–2.89)	2.87 (2.62–3.00)	2.80 (2.51–3.01)
Run_min*T*_-Ov1 (s)	2.68 (2.45–2.77)	2.73 (2.60–2.87); *p* = 0.030 *; ES = 0.36	2.75 (2.44–2.95); *p* = 0.058; ES = 0.26
Run_min*T*_-Ov2 (s)	2.61 (2.48–2.75)	2.77 (2.54–2.93)	2.78 (2.56–2.88)
Run_min*T*_-Ov3 (s)	2.63 (2.47–2.77)	2.78 (2.45–2.96); *p* = 0.018 *; ES = 0.47	2.74 (2.45–2.96); *p* = 0.063; ES = 0.27
RPE-Ov1	10 (8–12)	11 (10–14); ES = 0.79	11 (9–13); ES = 0.62
RPE-Ov2	12 (9–12)	12 (12–15); ES = 0.99	11 (10–14)
RPE-Ov3	12 (10–13)	12 (12–15); ES = 0.92	12 (11–15); ES = 0.62
Accuracy-Ov1 (%)	27.8 (11.1–37.5)	22.2 (16.7–37.5)	27.8 (0.0–43.0)
Accuracy-Ov2 (%)	38.9 (29.2–48.6)	22.2 (12.5–32.0); *p* = 0.028 *; ES = −0.39	30.6 (15.3–44.4); *p* = 0.080; ES = −0.27
Accuracy-Ov3 (%)	33.3 (19.4–45.8)	16.7 (9.0–26.4); ES = −0.93	27.8 (18.1–54.2)
Averaged over three overs			
Ball*_avgV_*-Avg (km h^−1^)	101.1 (95.1–108.7)	98.4 (94.6–102.3); *p* = 0.012 *; ES = −0.46	101.6 (96.2–104.1); *p* = 0.263; ES = −0.21
Ball*_peakV_*-Avg (km h^−1^)	104.7 (99.6–104.1)	103.2 (99.1–107.8); *p* = 0.012 *; ES = −0.56	105.9 (100.6–110.0); *p* = 0.441; ES = −0.14
Run*_avgT_*-Avg (s)	2.71 (2.51–2.81)	2.84 (2.62–3.00); *p* = 0.021 *; ES = 0.48	2.76 (2.56–2.88); *p* = 0.036 *; ES = 0.48
Run_min*T*_-Avg (s)	2.63 (2.47–2.77)	2.78 (2.56–2.94); *p* = 0.018 *; ES = 0.44	2.74 (2.45–2.96); *p* = 0.063; ES = 0.47
RPE-Avg	11 (10–13)	12 (12–15); *p* = 0.050 *; ES = 0.98	11 (11–14); *p* = 0.123; ES = 0.64
Accuracy-Avg (%)	31.9 (25.0–44.0)	27.8 (15.1–31.0); ES = −0.70	33.4 (18.5–41.7)

Ball*_avgV_*-Ov1, Ball*_avgV_*-Ov2, Ball*_avgV_*-Ov3—average ball velocity during first, second and third over; Ball*_peakV_*-Ov1, Ball*_peakV_*-Ov2, Ball*_peakV_*-Ov3—peak ball velocity during first, second and third over; Run*_avgT_*-Ov1, Run*_avgT_*-Ov2, Run*_avgT_*-Ov3—average run up times during first over, second and third over; Run_min*T*_-Ov1, Run_min*T*_-Ov2, Run_min*T*_-Ov3—minimum run up times during first over, second and third over; RPE-Ov1, RPE-Ov2, RPE-Ov3—average rating of perceived exertion during the first, second and third over; Accuracy-Ov1, Accuracy-Ov2, Accuracy-Ov3—accuracy of bowling during the first, second and third over; Ball*_avgV_*-Avg—average ball velocity averaged across the three overs; Ball*_peakV_*-Avg—peak ball velocity averaged across the three overs; Run*_avgT_*-Avg—average run up times averaged across the three overs; Run_min*T*_-Avg—minimum run up time averaged across the three overs; RPE-Avg—RPE averaged across the three overs; Accuracy-Avg—accuracy averaged across the three overs. * Significantly different from Tbase. Note: *p*-values have been provided only when the Friedman test exhibited a main effect time based on the paired Wilcoxon test, and ES have only been presented when a main effect of time was identified, or if the differences were moderate to large.

## Data Availability

The data presented in this study are available on request from the corresponding author.
